# Supplementary material for: Mars planetary insights and design framework for future in-situ aerial robotic missions
Source: Commun Eng. 2026 May 19;5:96. doi: 10.1038/s44172-026-00647-y (PMC13187032; doi:10.1038/s44172-026-00647-y)
Supplement: Supplementary file 2 — Supplementary Information - Notes [file 44172_2026_647_MOESM2_ESM.pdf]

Supplementary Information

Mars Planetary Insights and Design Framework for Future In-Situ Aerial Robotic Missions

Vishal Youhanna[1], Dmitry Ignatyev, Leonard Felicetti

Faculty of Engineering and Applied Sciences, Cranfield University, Cranfield, UK

This file includes:

1. Supplementary Note SN1 – Martian Radiation Effects

2. Supplementary Note SN2 – Martian Solar Irradiance

Note: References cited in this Supplementary Information document follow the numbering of the main manuscript reference list.

1 Corresponding Author.

Email address: vishal.youhanna@cranfield.ac.uk

Supplementary Notes

1. Supplementary Note SN1 – Martian Radiation Effects

This supplementary section provides the complete dataset and mechanisms underpinning the main-text summary in Section 3.2.4 Martian Radiation Effects.

Radiation, the emission of energy as electromagnetic waves or moving subatomic particles, is a fundamental aspect of space environments that affects Mars, comprises particle radiation and electromagnetic radiation [72], [75]. Particle radiation includes energetic particles that can penetrate deep into materials and biological tissues, such as the regular high-energy Galactic Cosmic Rays (GCRs) from outside the solar system, and the comparatively less energetic Solar Energetic Particles (SEPs) from the Sun, which occur irregularly during solar storms events [72]. The Sun experiences an approximately 11-year solar cycle that affects Mars' radiation exposure, with solar maximum increasing SEPs and suppressing GCRs due to the stronger solar magnetic field, while solar minimum allows more GCRs to reach the surface, increasing radiation levels [76]. Electromagnetic radiation spans a broad spectrum, including the damaging ultraviolet (UV) light, which primarily affects surfaces over prolonged time [75], [77]. On Earth, the ozone layer filters out UV radiation, while the planet's magnetic field protects against particle radiation [14]. Mars, lacking these protective shields [14], is exposed to higher levels of harmful radiation, compounding the challenges for unmanned and manned missions, including material degradation, electronic failures, and increased health risks for human explorers [72], [75], [77].

Radiation poses significant risks to spacecraft electronics and materials, leading to effects such as Total Ionizing Dose (TID), displacement damage, and Single Event Effects (SEE) [76]. TID results from cumulative ionization over time, causing shifts in voltage thresholds and increased leakage currents, which degrade the performance of electronic components. Displacement damage occurs when energetic particles displace atoms, creating defects that impair devices like solar cells. SEE involves a single radiation particle causing temporary errors or permanent damage in electronic circuits, ranging from minor glitches to complete system failures [76]. Ionizing radiation on Mars breaks down perchlorate salts into reactive compounds in the Martian regolith, accelerating corrosion in aluminium alloys commonly used in ground spacecraft by compromising protective oxide layers, especially when they are mechanically damaged [78]. Moreover, prolonged exposure to UV radiation can weaken the structural materials used in spacecraft, leading to a reduced lifespan of equipment [75], [77].

To mitigate radiation risks, spacecraft often use aluminium shielding, although its effectiveness is limited by factors like weight constraints and the potential generation of secondary particles from the shielding itself [76]. Advanced alternatives under development, such as hydrogen-rich carbon fibre composites and hydrogenated boron nitride nanotubes, aim to enhance radiation resistance and durability in space environments[79]. Radiation Hardening Assurance is the process of designing, selecting, and testing space electronics to ensure they can withstand the harsh radiation environment, incorporating strategies like using radiation-hardened components, redundancy, and shielding to maintain system reliability [80]. For example, NASA's Perseverance rover relies on specialised radiation-hardened components, such as the AD590S temperature sensor, which has been crucial in managing the extreme temperature variations experienced in space [81]. However, the trend toward using Commercial Off-The-Shelf (COTS) components, driven by their superior performance in terms of size, power, and speed, introduces new risks [80]. For instance, NASA's Ingenuity helicopter used a cell-phone grade processor, 150 times faster than Perseverance's processor, within a three-layer system [82]. It placed a radiation-tolerant FPGA at the base for low-level tasks, dual-redundant automotive-grade processors in the middle for flight control, and the Qualcomm Snapdragon 801 processor at the top for high-level functions, compensating for its lack of radiation hardening with redundant, parallel processing to ensure reliability [29]. As spacecraft electronics become more complex, advanced testing methods using higher energy particle beams and precise irradiation are crucial for developing hardened components that can withstand intense radiation environments [80]. Consequently, designing spacecraft for Mars necessitates robust shielding, materials resistant to UV degradation, and electronics that can endure high-energy particles to ensure mission success and longevity on the Martian surface.

Beyond uncrewed aerial systems, there have been conceptual studies such as in Ref. [83] and [84], exploring the feasibility of manned Martian rotorcraft. In such scenarios, radiation exposure becomes a critical factor in vehicle design, influencing shielding requirements, mission duration, and operational altitude constraints. The

absorbed dose of particle radiation is defined as the absorption of one joule of energy per kilogram of matter, measured in unit gray (Gy) [85]. To account for varying biological effects of different particle types, the absorbed dose is adjusted to does equivalent rate expressed in sieverts (Sv), reflecting the potential damage to living tissues [85]. A cumulative 1 Sv radiation exposure increases fatal cancer risk by 5%, which motivated NASA to set a 0.6 Sv astronaut career limit, while ESA and RSA set 1 Sv limits[86]. The average dose equivalent rate measured by instrument on the Curiosity rover at Gale Crater on Mars ranges from 0.64 to 0.721 milli-Sv/day, based on the respective solar minimum and maximum [85]. For comparison, astronauts on the ISS are exposed to about 0.5 mSv/day (182.5 mSv/year), as Earth's magnetic field offers some protection, whereas the average background radiation on Earth is only 0.007 mSv/day (or 2.4 mSv/yr) [85]. For a typical round-trip Mars mission, including 180 days each way and 500 days on the Martian surface, the total radiation dose could exceed 1 Sv, mainly because during their cruise to Mars, astronauts are exposed to radiation levels of approximately 1.84 mSv/day due to limited shielding in deep space [85]. Estimates from NASA's Mars Odyssey orbiter suggest that global surface radiation on Mars ranges from 100 to 200 mSv/year, with the lowest levels found at lower elevations where the thicker atmosphere offers more shielding (Fig. 3.913) [87]. These values are crucial for determining aerobot shielding thickness for safer radiation levels [85], particularly in systems designed for manned missions or biological payloads. Operating in lower Martian elevations with natural atmospheric shielding can further enhance protection.

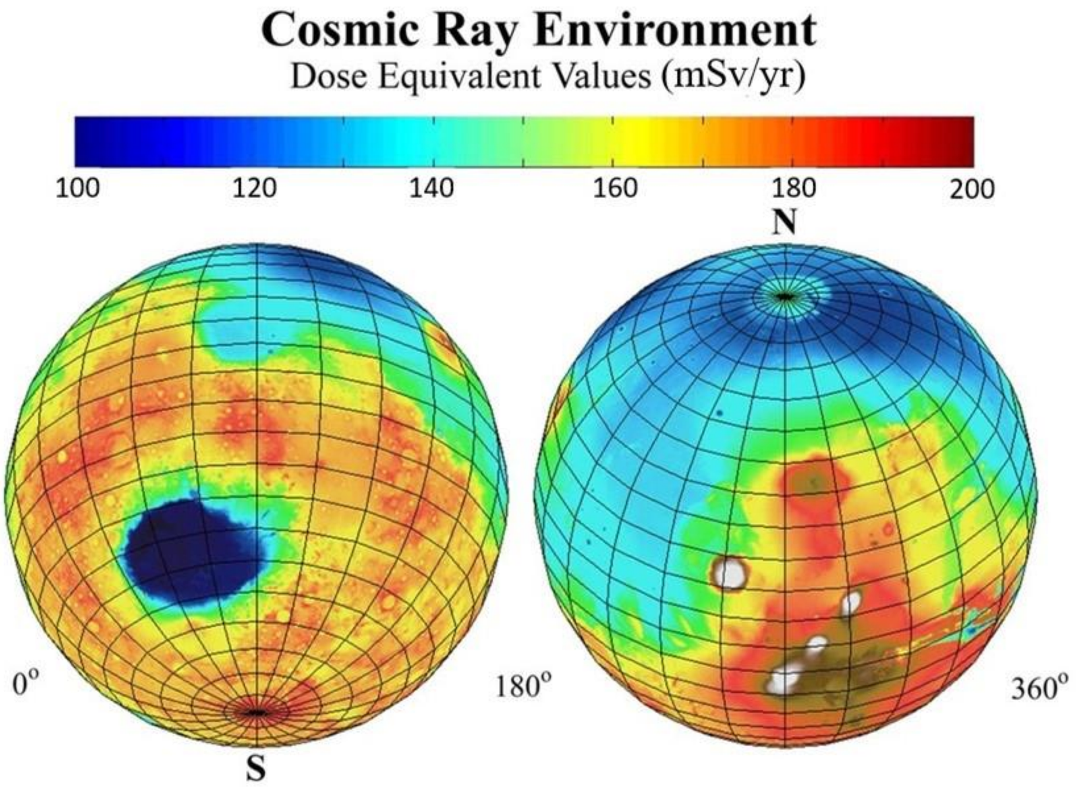

Fig.1 Estimated Radiation Dosage on Mars. The colours in the map refer to the annual dose equivalent in milli sievert (mSv) estimated utilising NASA's Mars Odyssey orbiter data. The range is generally from 100 Sv (colour-coded dark blue) to 200 Sv (colour-coded dark red) [87]. Original Image Credit: NASA/JPL/JSC. Modification: Altered equivalent units.

2. Supplementary Note SN2 – Martian Solar Irradiance

Solar irradiance, also referred to as solar flux, is the power per unit area received from the Sun in the form of electromagnetic radiation. The Martian solar constant (I_0), defined as the average solar irradiance received at the top of the atmosphere, is about 590 W/m² [88], about 57% [51] lower than that of Earth due to Mars' greater distance from the Sun. The mean irradiance reaching the Martian surface is often much lower due to atmospheric dust, which absorbs and scatters sunlight. This effect is quantified by the atmospheric optical depth (τ), ranging from 0.5 in clear conditions to over 3.0 during severe dust storms [88]. In a clear atmosphere, direct straight sunlight is vital for maximizing energy capture. The direct solar irradiance on a surface that is perpendicular to the Sun's rays ($I_\perp$) is given by [88]:

$$I_\perp = I_0 \exp\left(-\frac{\tau}{\cos\theta_z}\right), \tag{2.1}$$

where I_0 $(\mathrm{W/m^2})$ is the solar constant, τ is the atmospheric optical depth, and θ_z (degree°, solar zenith angle) is the angle between the Sun and the vertical direction above the surface point.

During dust storms, when direct sunlight is minimal, diffuse irradiance, which is sunlight scattered by atmospheric particles, becomes important as it reaches the surface from all directions. Global irradiance measures the total available solar energy by combining both direct and diffuse components along with surface-reflected sunlight [88]. The solar global irradiance on a horizontal surface at ground level on Mars can reach up to 400 W/m² at midday during a clear summer day and drop to as low as 80 W/m² at noon during a winter dust storm [88]. This measure is especially relevant for optimizing energy strategies and ensuring reliable power supply for solar-powered Mars aerobot systems, which might not always be oriented directly towards the Sun. Mars Climate Database (MCD) software [63] can be used to incident solar flux data in any region, space and time on Mars.

2.1 Photovoltaic Systems

Photovoltaic (PV) systems, which convert sunlight directly into electricity using solar cells, are particularly suitable for Martian aerobots due to being much lighter compared to nuclear power systems used in rovers. Maximising solar panel efficiency on Mars involves optimizing tilt and orientation [88]. Near the equator, lower tilt angles are effective, while steeper angles are necessary closer to the poles. Panels can be fixed or equipped with tracking systems. Fixed panels are simpler but less efficient, while tracking systems, which adjust panel orientation to follow the Sun's movement, offer higher energy capture but greater complexity [88]. Flat-plate solar arrays, a common type of PV system, are particularly effective as they can capture both direct and diffuse radiation, providing reliable power even during dust storms when diffuse light dominates [88]. In contrast, solar concentrators PV system, which uses reflectors to focus sunlight, are more efficient under clear conditions but are highly susceptible to the scattering effects of Martian dust, making them less ideal for consistent energy production [88].

Third-generation advanced thin-film III-V multi-junction solar cells, made from stacked materials in groups III and V of the periodic table, are used in space and Mars missions due to their broader spectrum capture, superior efficiency, radiation resistance, and lightweight flexibility, compared to the first-generation Crystalline Silicon cells predominantly used on Earth [89], [90]. Ingenuity Helicopter was equipped with Inverted Metamorphic Quadruple Junction solar cells [29], capable of efficiencies up to 33% [91]. Ongoing research aims at the theoretical 68.2% efficiency limit [90] for multijunction cells under standard sunlight, with the current record at 39.5% [92]. Interestingly, the significantly lower ambient temperatures on Mars reduce thermal losses and improve the overall energy conversion efficiency of solar cells; for instance, the efficiency of the Gallium Arsenide component in III-V multi-junction cells increases by about 10-30% compared to their performance on Earth [88], [93]. The following simplified equation can estimate the generic electrical power output (P_{el}) of the PV solar panel:

$$P_{\mathrm{el}} = \eta I_{\mathrm{G}} A\,, \tag{2.2}$$

where I_G $(\mathrm{W/m^2})$ is the global solar irradiance $(= I_{\mathrm{direct}} + I_{\mathrm{diffuse}} + I_{reflected})$ [88], A $(\mathrm{m^2})$ is the area of solar panel, and η is the overall efficiency of the solar cell (includes losses due to factors like conversion inefficiencies, reflection, and thermal effects).

For future aerobot missions, reliability is paramount. Despite their resistance, III-V solar cells degrade over long missions on Mars due to cumulative radiation exposure, which displaces atoms within the lattice and causes defects, as well as thermal and mechanical stress, and material ageing [90]. However, dust deposition is the major issue, significantly affecting the lifetime, maintenance, and energy output of solar power systems, as evidenced by the 0.28% performance degradation per sol observed on the Pathfinder solar arrays [88], [93]. The findings from the long-term Mars Exploration Rovers (MER) mission suggest that the degradation rate decreased to about half as additional dust layers had a diminishing impact [93]. While winds can temporarily remove dust, leading to reversible effects, the study [94] on MER-B found an irreversible degradation rate of 0.6% per year due to cell cracking, surface abrasion, and interconnect failures, resulting in a 1.9% annual power loss. The most severe damage comes from global dust storms, equivalent to approximately 77 sols of standard exposure [94]. Although various dust mitigation technologies exist, they are excluded from spacecraft designs due to trade-offs in complexity, power consumption, weight, and limited effectiveness [94]. Mechanical wipers, like those used on Earth, physically sweep dust off surfaces but can cause surface abrasion over time, leading to reduced solar panel efficiency and potential material degradation. Air-blast cleaning systems rely on high-pressure gas to dislodge dust, but in Mars' low-pressure atmosphere, the reduced air density limits their effectiveness. Electrostatic dust removal systems use electric fields to repel or move dust particles, a promising technology; however, they require additional power, precise surface integration, and complex control mechanisms, making them less practical for current Martian aerobot designs [95].

To ensure the long-term efficiency of solar panels on Martian aerobots, practical strategies must be cautiously integrated with mission constraints. System redundancy mitigates power loss by incorporating multiple energy sources or backup panels. Real-time data monitoring allows dynamic power adjustments based on dust accumulation trends. Durability testing ensures materials can withstand repeated dust exposure over extended missions. Anti-reflective coatings reduce dust adhesion, improving light absorption, while optimising panel tilt angles (45°–60°) enhances passive dust shedding by leveraging Martian wind dynamics and gravity [94]. This comprehensive approach to managing solar flux is crucial for the success and longevity of solar-powered aerobot missions in Mars' challenging environment.
