# Supplementary material for: Mars planetary insights and design framework for future in-situ aerial robotic missions
Source: Commun Eng. 2026 May 19;5:96. doi: 10.1038/s44172-026-00647-y (PMC13187032; doi:10.1038/s44172-026-00647-y)
Supplement: Supplementary file 3 — Description of Additional Supplementary Files [file 44172_2026_647_MOESM3_ESM.pdf]

Description of Additional Supplementary Files:

File: Supplementary Information – Notes

Description: This PDF contains supplementary notes supporting the main manuscript. Supplementary Note S1 summarises Martian radiation environment considerations, including electronic effects and shielding context. Supplementary Note S2 summarises solar irradiance, photovoltaic design considerations, degradation mechanisms and dust-mitigation context. The file also includes one supplementary figure and uses the reference numbering of the main manuscript.

File: Supplementary Data

Description: This Excel workbook contains the aerobot catalogue dataset compiled from the literature and used to inform this Perspective. It is organised across six sheets and provides a structured survey of Mars aerobot concepts, including concept classification and related descriptive information. The spreadsheet format allows filtering and navigation.
